# Advantages of immediate implant-based breast reconstruction over delayed breast reconstruction in women treated with postmastectomy radiotherapy for breast cancer

**DOI:** 10.1007/s10549-025-07690-x

**Published:** 2025-05-12

**Authors:** Merel M. L. Kooijman, J. Joris Hage, Astrid N. Scholten, Frederieke H. van Duijnhoven, Corstiaan C. Breugem, Leonie A. E. Woerdeman

**Affiliations:** 1 Department of Plastic and Reconstructive Surgery, Netherlands Cancer Institute-Antoni van Leeuwenhoek Hospital, Plesmanlaan 121, 1066 CX Amsterdam, Noord-Holland The Netherlands; 2 Department of Radiotherapy, Netherlands Cancer Institute-Antoni van Leeuwenhoek Hospital, Amsterdam, The Netherlands; 3 Department of Surgical Oncology, Netherlands Cancer Institute-Antoni van Leeuwenhoek Hospital, Amsterdam, The Netherlands; 4 Department of Plastic and Reconstructive Surgery, Amsterdam University Medical Center, Amsterdam, The Netherlands

**Keywords:** Breast cancer, Mastectomy, Breast reconstruction, Breast implant, Radiotherapy

## Abstract

**Purpose:**

To compare immediate with delayed breast reconstruction in the setting of postmastectomy radiotherapy (PMRT) in terms of the total number of interventions and time required for breast cancer clearance and contour restoration.

**Methods:**

The long-term prevalence and number of plannable and urgent interventions required in women receiving PMRT to finish 372 nipple-sparing or skin-sparing mastectomies combined with immediate implant-based breast reconstruction ([N]SSM/IIBR) were compared to those required for 18 mastectomies and delayed breast reconstruction (DBR) performed between 2013 and 2019.

**Results:**

Re-interventions were required in 239 of the 372 breasts (64%) after [N]SSM/IIBR, whereas all 18 DBRs (100%) implicitly required at least one re-intervention (*p* < 0.001). Mastectomy and reconstruction necessitated a mean of 2.24 interventions per breast after [N]SSM/IIBR, which was significantly less than the mean of 3.72 interventions per breast after DBR (*p* < 0.001). Breast contour reconstruction was achieved in 14.3 months after [N]SSM/IIBR and in 38.6 months after DBR (*p* < 0.001). [N]SSM/IIBR required more class U3 urgent re-interventions than DBR (22% vs. 4%, *p* = 0.002), whereas DBR necessitated more class P3 plannable re-interventions (5% vs. 16%, *p* = 0.004). Initiation of PMRT is not postponed after [N]SSM/IIBR (10.1 weeks) compared to DBR (14.0 weeks).

**Conclusions:**

Women potentially needing PMRT should be informed pre-operatively that [N]SSM/IIBR with PMRT may be associated with 22% severe complications and 8% failure. Still, [N]SSM/IIBR prior to PMRT required less interventions and was less time-consuming than DBR following PMRT. Therefore, the potential need of re-interventions should not be the reason for refraining from [N]SSM/IIBR in these women.

## Introduction

Combined nipple-sparing or skin-sparing mastectomy ([N]SSM) and immediate breast reconstruction is esthetically superior to delayed breast reconstruction (DBR) because of the conservation of the recognizable skin envelope and inframammary fold. It provides better quality of life by immediate restoration of the feminine body contour, self-image, and sexuality [1, 2]. From the patient’s perspective, it, furthermore, is associated with less physical distress, time investment, and psychosocial stress because of the possibility to finish surgical treatment in one intervention [2, 3]. Some women, even, prefer sole mastectomy to breast reconstruction to avoid additional interventions [4]. In these women, immediate reconstruction may prevent permanent loss of contour as a result of treatment fatigue [2]. For logistic, technical, and morbidity reasons, the majority of such immediate breast reconstructions is implant-based rather than based on autologous tissue transplantation [1, 2, 4].

Immediate implant-based breast reconstruction (IIBR) in the setting of postmastectomy radiotherapy (PMRT) remains controversial, as it can potentially be associated with two main problems [4–7]. First, PMRT may increase the postoperative complication rate and worsen the esthetic outcome of [N]SSM combined with IIBR and, second, an eventful outcome of combined [N]SSM/IIBR may delay the initiation of PMRT [5].

Previously, we showed that PMRT is not associated with a higher number of short-term and long-term complications following [N]SSM/IIBR and that [N]SSM/IIBR does not delay PMRT initiation [8, 9]. To date, however, we did not assess whether immediate or delayed reconstruction is preferred in the setting of PMRT in terms of prevalence of long-term complications and re-interventions. Such an assessment is indicated because it offers the patient a perspective on the total number of interventions that may be required for breast cancer clearance and breast contour restoration. As such, it may enhance reaching a shared decision regarding the treatment. Therefore, we compared the prevalence and number of interventions required to finish [N]SSM/IIBR to those required for mastectomy and DBR, in women who received PMRT.

## Methods

### Patients

#### Immediate reconstruction group and delayed reconstruction group

Since January 1st, 2013, we routinely offer [N]SSM/IIBR to all women presenting with primary breast cancer irrespective of an indication for PMRT. Between 2013 and 2019, 372 breasts in 370 such women were treated in our institute with PMRT after combined [N]SSM/IIBR and could be included in the *immediate reconstruction* group (IR group) for analysis. In the same period, another 18 women underwent sole mastectomy and PMRT for primary breast cancer, which was followed by delayed reconstruction only after completion of oncologic treatment (*delayed reconstruction group*, or DR group). We previously reported why some women planned to undergo PMRT forgo immediate breast reconstruction mainly because of patient preference [10]. These women are offered a postoperative consultation with the plastic surgeon to discuss delayed breast reconstruction.

### Surgical treatment and PMRT

#### Mastectomy, immediate implant-based breast reconstruction, and PMRT

All 372 breasts in the IBR group were operated on in a standardized fashion [9, 11]. Skin-sparing mastectomy was performed in 215 breasts (58%) and nipple-sparing mastectomy was performed on the other 157 breasts (42%). The mastectomy was combined with a sentinel node procedure in 233 breasts (63%) and with immediate axillary lymph node dissection in 100 breasts (27%).

Immediately following [N]SSM, a direct-to-implant approach (*n* = 356, or 96%) was performed using a textured, high-cohesive gel-filled permanent prosthesis (Natrelle style 410; Allergan®) implanted in a subpectoral dual-plane position. Prepectoral implantation was not applied in this series because we were not yet convinced of its advantages by the preliminary results available on this technique, during the inclusion period of the study (2013–2019). For a two-staged approach (*n* = 16, or 4%), a saline solution-filled tissue expander was inserted (Natrelle style 133; Allergan®). No acellular dermal matrix or synthetic mesh was used. Antibiotic prophylaxis and wound drainage were applied in all women. PMRT started at a mean of 10.1 weeks after mastectomy, with a median dose of 4256 cGy given in 10 to 25 fractions. Mean follow-up after combined [N]SSM/IIBR was 54.6 months (Table 1).

**Table 1:** Mean (range; standard deviation) or number (percentage) of baseline characteristics among our study groups

Characteristic	Immediate breast reconstruction	Delayed breast reconstruction	*p* value
	(*n* = 372)	(*n* = 18)	
Age [years]^a^	44.7 (22–72; 9.81)	43.8 (25–61; 10.0)	0.651
BMI [kg/m^2^]^a^	24.1 (16.7–43.4; 3.77)	26.5 (17.2–41.9; 6.14)	0.098
Comorbidity^b^	49 (13%)	7 (39%)	**0.008**
Tobacco use^c^	26 (7%)	3 (17%)	0.141
Previous BCS^d^	33 (9%)	2 (11%)	0.670
Invasive carcinoma	367 (99%)	18 (100%)	1.000
Advanced stage breast cancer^e^	192 (52%)	13 (72%)	0.096
Contralateral surgery^f^	68 (18%)	0 (0%)	0.052
PMRT initiation^g^	10.1 (2–72; 8.04)	14.0 (4–35; 9.57)	0.172
PMRT dosage [cGy]^h^	4256 (3000–5000)	4256 (3000–5000)	0.100
Follow-up^i^	54.6 (3–102; 22.02)	53.3 (7–84; 25.65)	0.980
Reconstruction type			
Direct-to-implant	356 (96%)	n.a	–
Tissue expander (two-staged)	16 (4%)	1 (6%)	–
DIEP reconstruction	n.a	10 56%)	–
Hybrid reconstruction (LD flap)	n.a	6 (33%)	–
Hybrid reconstruction (TD flap)	n.a	1 (6%)	–

Statistically significant *p* values are provided in bold print

*PMRT* postmastectomy radiotherapy, *DIEP* deep inferior epigastric perforator, *LD* latissimus dorsi, *TD* thoracodorsal

^a^Mean of women instead of breasts

^b^Diabetes mellitus, obesity, cardiovascular, pulmonary or hematologic disorder, and/or rheumatoid arthritis

^c^Negative in case patient stopped smoking > 6 months pre-operative

^d^BCS = breast-conserving surgery without (neo-)adjuvant radiotherapy

^e^Invasive carcinoma stage IIIA or higher

^f^Initial reconstructive procedure combined with simultaneous contralateral breast surgery

^g^Mean PMRT initiation in weeks after mastectomy

^h^Median (with range) PMRT dosage in Grays

^i^Follow-up (in months) since initial reconstructive intervention

#### Mastectomy, PMRT, and delayed breast reconstruction

Total mastectomy including the nipple–areolar complex and surplus skin was performed in all 18 breasts in the DBR group. Mastectomy was combined with a sentinel node procedure in 6 of the 18 breasts (33%) and with immediate axillary node dissection in 11 breasts (61%). Antibiotic prophylaxis and wound drainage were applied in all women. PMRT started a mean of 14.0 weeks after the mastectomy, with a median radiation dose of 4256 cGy, also given in 10 to 25 fractions.

Secondary breast reconstruction started on average 22.0 months (range, 10—59 months; SD, 13.73) after mastectomy by means of implantation of a tissue expander (*n* = 1), a hybrid technique using a thoracodorsal flap (*n* = 1), or latissimus dorsi flap (*n* = 6) in combination with a tissue expander [12, 13], or microsurgical DIEP free flap (*n* = 10) transplantation [14]. Mean follow-up after initiation of delayed reconstruction was 53.3 months (Table 1).

### Data gathering and analysis

#### Baseline characteristics

Data on patient-related and treatment-related characteristics that may act as risk factors for an eventful outcome of breast reconstruction were retrieved from a prospectively maintained digital database. These included age, smoking habits, breast cancer stage, and PMRT (Table 1 and 2). Locally advanced stage breast cancer was defined as stage IIIA or higher [15, 16]. As we previously found that neoadjuvant or adjuvant chemotherapy does not significantly influence the outcome of breast reconstruction, we did not assess these as separate risk factors [9, 26].

**Table 2:** Distribution of breast cancer stages among both study groups

Breast cancer stage	Immediate breast reconstruction		Delayed breast reconstruction		*p* value
	(*n* = 372)		(*n* = 18)		
	*n*	%	*n*	%	
Stage I	10	3%	0	0%	1.000
Stage IIA	75	20%	3	17%	1.000
Stage IIB	88	24%	2	11%	0.267
Stage IIIA	113	30%	9	50%	0.115
Stage IIIB	57	15%	3	17%	0.747
Stage IV	22	6%	1	6%	1.000
Non-staged^a^	7	2%	0	0%	1.000

^a^DCIS (*n* = 5), phyllodes tumor (*n* = 1), and a solitary metastasis of lung carcinoma (*n* = 1)

#### Outcome measures

The primary outcome measures were the number of breasts requiring re-interventions, the mean number of (re-)interventions per breast, and the time needed for breast cancer clearance and breast contour restoration [17, 18]. Only interventions requiring surgery under general anesthesia were assessed as objective benchmarks for clinically significant events. This study was performed from the patient’s perspective on the total number of interventions. Therefore, this number of interventions comprised the [N]SSM/IIBR procedure in the IR group, the mastectomy and secondary reconstruction in the DR group, and all plannable and urgent surgical re-interventions on the reconstructed breast during follow-up in both groups [7, 19, 20]. *Plannable re-interventions* were defined as expected and elective components of the reconstruction process and included second-stage procedures to replace a temporary expander, correction of capsular contraction to be expected after PMRT, and long-term cosmetic breast mound enhancements such as scar revision or lipofilling. Furthermore, we included the replacement of an implant for autologous tissue. *Urgent re-interventions* were defined as revisions resulting from acute complications such as hematoma, abscess or skin necrosis, and vascular re-interventions (Clavien Dindo class 3b) [21].

**Fig. 1:**
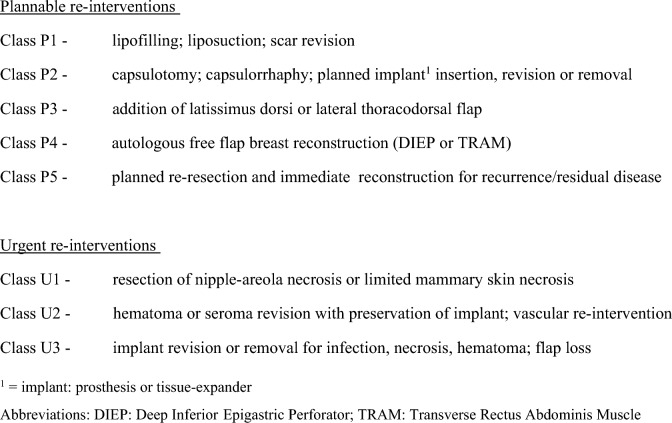
Classification of plannable and urgent re-interventions according to surgical impact

As secondary outcome measures, both the plannable and the urgent re-interventions were classified according to severity (Fig. 1) and the frequency of implant replacements and reconstruction failure were recorded. Reconstruction failure was defined as a lack of reconstructed breast contour at the end of our study period. Nipple reconstruction and areolar tattooing were not scored as separate interventions as they are routinely done under local anesthesia [19, 22].

#### Statistical analysis

The distribution of baseline characteristics between both groups was compared using the Mann–Whitney U-test for continuous variables, and the Fischer exact test for dichotomous variables.

The fraction of breasts requiring at least one surgical intervention after mastectomy, the fraction of replacements and reconstruction failure, and the fraction of plannable and urgent interventions in both groups were compared using the Fischer exact test. The Mann–Whitney U-test was used to compare the groups in terms of the mean number of (re-)interventions required per breast and the mean time required for breast cancer clearance and contour restoration. These statistical tests were used to correct for the small number of breasts in the DR group and difference in sample size between both groups. All statistical analyses were performed using SPSS (Version 29.0; SPSS Inc., Chicago, Illinois, United States) and *p* values of 0.05 or less were accepted as statistically significant.

## Results

### Baseline characteristics

All patient- and treatment-related characteristics, including PMRT dose, of both groups were comparable except for the fraction of comorbidities (Tables 1 and 2).

### Outcome measures

**Fig. 2:**
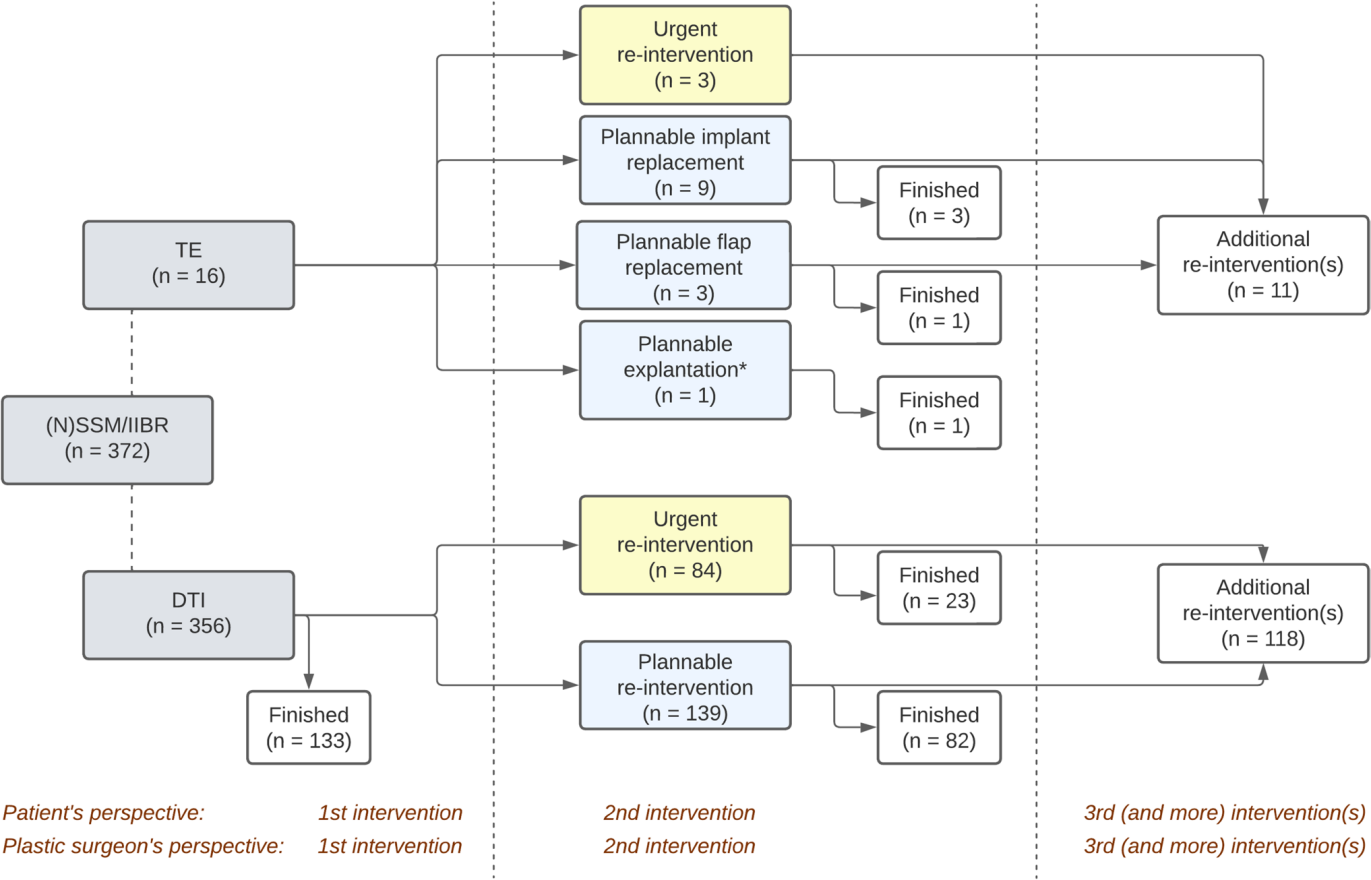
**Interventional paths of 372 immediately reconstructed breasts following nipple- or skin-sparing mastectomy**. Distribution of 372 immediately reconstructed breasts over the various interventional paths. Note that the 16 women in whom a tissue expander was implanted during [N]SSM/IIBR implicitly required one re-intervention to replace the tissue expander, whereas 133 women who underwent a direct-to-implant immediate reconstruction had their reconstruction finished in one intervention. Furthermore, note that, in this setting, the patient’s perspective matches the plastic surgeon’s perspective on the required number of interventions. *TE* tissue expander, *DTI* direct-to-implant; *: explantation by choice of patient

**Fig. 3:**
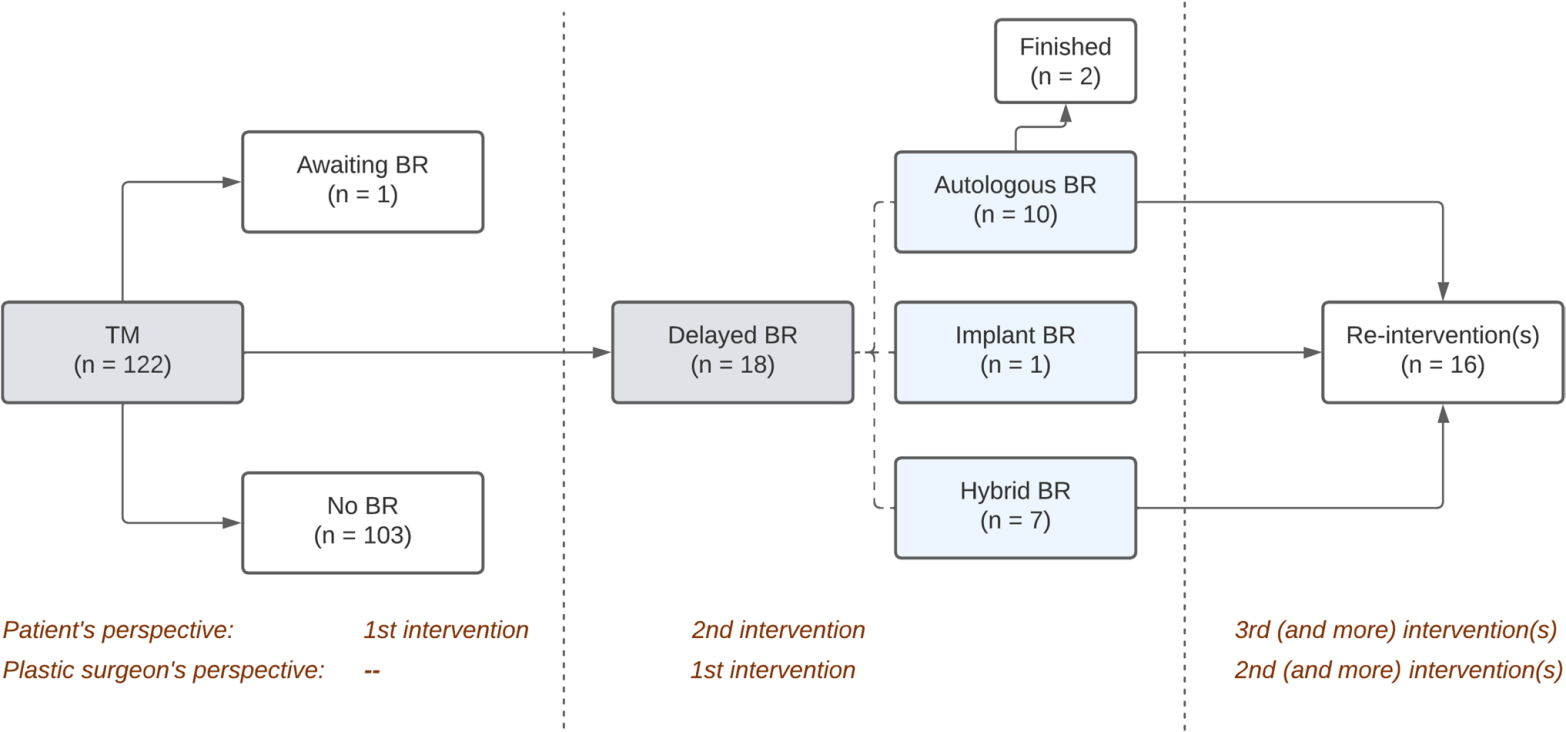
**Interventional paths of 122 total mastectomies that were not reconstructed immediately.** Distribution of the 122 breasts that were not immediately reconstructed during the total mastectomy, over the subsequent treatment options. Note that from the patient’s perspective, women in the delayed reconstruction group implicitly undergo one operation that is ignored in the plastic surgeon’s perspective: the initial mastectomy. *TM* total mastectomy, *BR* breast reconstruction

Re-interventions under general anesthesia were required in 239 of the 372 breasts (64%; 95% CI 59–69%) after [N]SSM/IIBR (Fig. 2). In the DR group, all 18 reconstructions (100%; 95% CI 87–100%) implicitly required at least one re-intervention after the mastectomy (*p* < 0.001) (Fig. 3). Likewise, all tissue expander-based [N]SSM/IIBR implicitly required at least one re-intervention, but 133 out of the 356 permanent prosthesis-based [N]SSM/IIBR (37%) only needed the initial combined procedure to finish breast reconstruction (Fig. 2).

A total of 832 interventions were required in the IR group (*n* = 372), or 2.24 (95% CI 2.10–2.37) interventions per breast (Table 3). This number was significantly less than the 67 interventions performed in the 18 DBR group, or 3.72 (95% CI 3.22–4.23) intervention per breast (*p* < 0.001). This difference reflected the difference between 693 plannable interventions among the 372 immediately reconstructed breasts (1.86 per breast; 95% CI 1.76–1.96), and 60 such interventions among the 18 delayed reconstructions (3.33 per breast; 95% CI 2.91–3.75; *p* < 0.001). Contrastingly, the fraction of 139 urgent interventions among the 372 IBRs (0.37 per breast; 95% CI 0.30–0.44) matched the 7 urgent interventions among the 18 delayed reconstructions (0.39 per breast; 95% CI 0.16–0.62; *p* = 0.92).

**Table 3:** Number (percentage) or mean number (range; standard deviation) of interventions, re-interventions, replacements, reconstruction failures, and time required for treatment among our study groups

	Immediate breast	Delayed breast	*p* value
	reconstruction	reconstruction	
	(*n* = 372)	(*n* = 18)	
Total number of interventions^a^	832	67	
Interventions per breast^b^	2.24 (1—10; 1.33)	3.72 (2—6; 1.02)	** < 0.001**
Breasts requiring re-intervention^c^	239 (64%)	18 (100%)	** < 0.001**
Number of re-interventions^c^	460	49	
Re-interventions per breast^d^	1.24 (0—9; 1.33)	2.72 (1—5; 1.02)	** < 0.001**
Number of replacements^e^	50 (13%)	0 (0%)	0.146
Reconstruction failure	30 (8%)	0 (0%)	0.381
Months required for treatment^f^	14.3 (0—92; 19.0)	38.6 (12—64; 16.8)	** < 0.001**

Statistically significant *p* values are provided in bold print

^a^Total of interventions required for mastectomy and breast reconstruction in that group

^b^Mean number of interventions per breast in that group

^c^Re-interventions after mastectomy (delayed group) or combined [N]SSM-IBR (immediate group)

^d^Mean number of re-interventions per breasts in that group

^e^Number of breasts in which the implant was fully replaced by an autologous flap

^f^Mean time span from (combined) mastectomy to final reconstruction intervention

Breast contour reconstruction was achieved in a mean of 14.3 months (95% CI 12.4–16.3) after [N]SSM/IIBR, and in a mean of 38.6 months (95% CI 30.6–47.0) after the mastectomy in the DR group (*p* < 0.001) (Table 3).

The distribution of re-interventions over the classes of severity indicates that the significant difference between the number of plannable interventions in both study groups may particularly be caused by the significantly higher prevalence of Class P3 capsulotomy, capsulorrhaphy, and plannable implant revision or removal in the DR group (5% vs 16%; *p* = 0.004) (Table 4). Capsular contracture led to re-interventions in 78 of the 372 breasts (21%) in the IR group and in 2 of the 18 breasts (11%) in the DR group.

**Table 4:** Number and percentage of plannable and urgent re-interventions according to class of surgical impact^a^ among both study groups

Class of re-intervention^a^	Immediate breast reconstruction		Delayed breast reconstruction		*p* value
	(*N* = 460 re-interventions)		(*N* = 49 re-interventions)		
	*n*	% (*n*/*N*)	*n*	% (*n*/*N*)	
Plannable re-intervention					
Class P1	124	27%	12	24%	0.865
Class P2	125	27%	12	24%	0.738
Class P3	21	5%	8	16%	**0.004**
Class P4	47	10%	10	20%	0.052
Class P5	4	1%	0	0%	1.000
Urgent re-intervention					
Class U1	15	3%	0	0%	0.382
Class U2	24	5%	5	10%	0.184
Class U3	100	22%	2	4%	**0.002**

Statistically significant *p* values are provided in bold print

^a^See Fig. 1 for the classification of re-interventions

Of the urgent re-interventions, the prevalence of Class U3 implant revision or removal for infection, necrosis, hematoma, and flap loss was significantly higher (22% vs 4%; *p* = 0.002) in the IR group (Table 4).

Fifty of the 372 implants (13%) were replaced for autologous tissue in the IR group, and latissimus dorsi or lateral thoracodorsal flaps were added for a hybrid reconstruction in 18 others (5%). Additionally, three latissimus dorsi or lateral thoracodorsal flaps were used for breast mound enhancement after partial fat necrosis (*n* = 1) or total flap failure (*n* = 2) of DIEP flaps used in this group. In 17 of the 18 breasts in the DR group (94%), a purely autologous (*n* = 10) or hybrid implant/autologous (*n* = 7) reconstruction was performed (Fig. 3).

Implant revision or removal for infection, necrosis, hematoma, or flap loss occurred 102 times in the total of 390 breasts during the study period (Table 4). At the end of the study period, a breast contour was achieved for all 18 breasts in the DR group (*p* = 0.381) whereas a reconstructed breast contour lacked in 30 of the 372 IBRs. Because we cannot be certain whether these women might still pursue secondary reconstruction at a later stage, we accepted this last number as reconstruction failures.

## Discussion

In this study, we found that a significantly lower percentage of breasts undergoing PMRT after the [N]SSM/IIBR required at least one re-intervention (64%), compared to those that were reconstructed after PMRT (100%; *p* < 0.001). Partly, this may be explained by the implicit additional need of the initial reconstructive procedure in the DR group following mastectomy, whereas this reconstructive procedure is combined in the same procedure as the mastectomy in the IR group. Even when the initial reconstructive procedure would be ignored in the DR group, however, the number of breasts requiring at least one re-intervention after [N]SSM/IIBR (64%) would still be significantly lower than that in the DR group (16/18 or 89%; *p* = 0.040). This holds true even though we observed significantly more Class U3 re-interventions after immediate reconstruction (100/460, or 22%) than after delayed reconstruction (2/49, or 4%). Class U3 re-interventions and the related complications may have caused physical and psychosocial distress to these women, and 8% of immediate reconstructions resulted in reconstruction failure.

Breast reconstruction often is a multistage process with rates of re-interventions after the initial reconstructive procedure reportedly varying from 37 to 88% [6, 7, 19, 22]. The Mastectomy Reconstruction Outcomes Consortium (MROC) reported that in 22 to 39% of the patients, at least one breast complication occurred within the first two years postoperatively, depending on the technique of reconstruction and whether or not they had radiotherapy [23]. In a Canadian population-based study, Roberts et al. [19] observed that three or more re-interventions were even required in 35% of 3972 patients during a mean follow-up of 5.1 years. The authors later reported at least one urgent re-intervention on the breast in 48% of 3,066 women who underwent immediate or delayed reconstruction [20]. This risk was comparable for both immediate and delayed reconstructions (*p* = 0.47), but they reported a significantly increased risk of re-interventions in case of PMRT (*p* < 0.001) [20].

Of main interest in terms of the number of interventions potentially required for a woman to rid herself of breast cancer and have her breast contour restored, we found that significantly less interventions per breast were needed to achieve mastectomy and breast reconstruction in the IR group. This reflects that significantly less plannable interventions were needed after [N]SSM/IIBR. Again, the observed higher number of plannable interventions in the DBR group can partly be explained by the implicit occurrence of an isolated mastectomy in this group with a secondary reconstruction as first re-intervention.

There is a lack of reports detailing the average number of operations required for breast reconstruction [20, 22], and even less is reported on the quantitative impact of PMRT on this number [7]. In 2014, Eom et al. [22] were the first to report a mean number of 2.34 (range, 1–9) of interventions required in a group of 182 immediate breast mound reconstructions. The mean number of interventions required among 72 delayed reconstructions was higher, but not significantly so (2.46; *p* = 0.69) [22]. Reporting from their plastic surgical perspective, however, the authors apparently did not record the initial mastectomy as an intervention in their DR group. Including this mastectomy to acknowledge the patients’ perspective would increase the mean number in their DR group to 3.46, which likely is significantly higher than the mean of 2.34 interventions in their IR group. These numbers match our observed averages of 3.72 per breast in our DR group and 2.24 in our IR group.

Roberts et al. [19] explicitly mentioned that they reported on the mean number of *re-*interventions after the initial breast reconstructive procedure. Again, no significant difference was observed between this mean number for immediate reconstruction (2.4) versus delayed reconstruction (2.3) from this plastic surgical perspective [19]. Because the authors did not consider the combined mastectomy and reconstruction in the IR group, nor the sole mastectomy and subsequent initial reconstructive procedure in the DR group as interventions, their reported numbers appear to reflect that significantly less interventions were required to finish combined mastectomy and immediate reconstruction than to finish mastectomy and subsequent delayed reconstruction.

We observed a permanent implant replacement rate of 13% and reconstruction failure rate of 8% in the IR group after 4.6 years of follow-up. These rates compare to the 7-year implant replacement rate of 17% and implant removal rate of 13% by Shumway et al. in 2020 [3].

### Potential limitations

As a potential methodological limitation, we note that there is a difference between group sizes. We included the data on all consecutively treated women in both groups even though this resulted in a much larger number of women in the IR group than in the DR group. This was done to prevent any potential selection bias of a case-matched control study, to obtain more power of our statistical analysis, and to prevent loss of valuable information. The number of women in the DR group is limited (*n* = 18) because our policy is to offer [N]SSM/IIBR, irrespective of possible PMRT indication. To correct for this limitation, we used Fisher exact and Mann–Whitney U-tests [24, 25]. The significant *p* values observed indicates that the difference between groups is real and substantial enough to be detected even in a limited sample. This is supported by the lack of overlapping confidence intervals of the primary outcome results. We, furthermore, restricted our assessment to women who were treated in one institute during a restricted period of time to minimize institutional and heterogeneity biases, as well as surgical performance biases [17]. As in any observational study, in general, selection bias may exist. As such, the significant difference in prevalence of comorbidities, a trend toward differences in BMI and more advanced stage cancers, and a difference in rate of autologous reconstruction might have influenced the observations in this study. Although randomization would overcome this pitfall, this cannot be accomplished in breast reconstruction given the oncological and preferential considerations of each patient, and institutional practice patterns for the choice of reconstruction.

Second, we did not assess the distribution of (neo-)adjuvant chemotherapy as a risk factor because we previously found no difference in surgical complications between women undergoing (neo)adjuvant chemotherapy and those who did not [26, 27]. We, furthermore, observed that the pre- and postoperative oncological characteristics we assessed (fraction of carcinomas, tumor stage, PMRT dosage, etcetera) did not significantly differ between the IR group and the DR group.

Third, we only scored the interventions performed on the reconstructed breast. Autologous flap donor site re-interventions or symmetrizing procedures to the contralateral breast were not assessed. We did so to prevent confusion regarding the definition of procedures to be included. As such, previous reports on re-interventions have been inconsistent as in some patients such procedures were scored as an additional intervention, while in other patients, they were not [7, 19, 20, 22]. Furthermore, such secondary re-interventions may not been scored because they were performed in other hospitals, leading to an underestimation of the re-intervention rates [7].

Last, our study was not designed to explore causative differences between immediate implant-based and delayed autologous reconstruction and the relatively small DR group would have underpowered any analysis in that regard [17]. Future analysis is needed to elicit the factors contributing particularly to urgent reoperations, as these are a relevant target for quality improvement [19].

### Clinical implications of our observations

We prefer to perform [N]SSM/IIBR in women presenting for a primary mastectomy. Preservation of the mammary integumental architecture reduces the number of re-interventions and improves the cosmetic appearance of the breast, thereby diminishing the need for secondary skin flap addition or expansion and remodeling after PMRT [4]. We routinely perform a subpectoral implantation because autologous reconstruction is associated with an additional donor site and an extended, more complicated procedure with a longer recovery time [2, 4, 28]. This way, we have been able to ensure that adjuvant treatment is not postponed by possible complications of the combined procedure [8, 27]. In case the patient persists in her preference for autologous reconstruction, we will replace the implant for a flap only after any adjuvant therapy has been completed [4]. This approach offers our patients the advantage of preventing any time without a breast and finishing their reconstruction in significantly less time than required to finish a DBR (14.3 vs. 38.6 months; *p* < 0.001). With it, we achieve a relatively high ratio of 83% of IBRs among women who undergo mastectomy [9]. Our first alternative is to delay an autologous reconstruction until after completion of mastectomy and PMRT in order to prevent radiation damage to the autologous flap.

The number of interventions needed often exceeds the number expected. An unexpected number of additional interventions required after mastectomy and reconstruction may well lead to increase of postsurgical morbidity, decrease of patient satisfaction, and reduction of the patient’s quality of life [7, 19]. Therefore, women should be actively informed on the number of operations that might be required when they make their reconstruction choices at the beginning of oncological treatment [22]. Choosing between immediate or delayed reconstruction in the setting of PMRT remains complex and necessitates shared decision-making. This process also requires detailed information on the long-term complications and re-interventions with both procedures to provide patients a perspective on the total number of interventions needed for cancer clearance and contour restoration.

We have now shown that combined [N]SSM/IIBR in the setting of PMRT is favorable in terms of the total number of interventions and time investment a woman needs for the oncological and reconstructive treatment. Even though PMRT may lead to clinically relevant risks of plannable and urgent re-interventions and, even, reconstructive failure after [N]SSM/IIBR, the majority of these patients ultimately retain their implant-based reconstruction with only a minority of patients requiring conversion to an autologous reconstruction [3, 6]. Furthermore, we confirmed that immediate breast reconstruction does not delay start of PMRT. Still, the results of our study should not be interpreted as immediate implant-based reconstruction to be *superior* to delayed autologous reconstruction for women requiring PMRT. Rather, we recommend an open and individualized approach to all patients in deciding on their optimal strategy [1].

In conclusion, women who might be facing PMRT should pre-operatively be informed that [N]SSM/IIBR with subsequent PMRT was associated with 22% severe complications and 8% reconstruction failure. Still, [N]SSM/IIBR prior to PMRT required less interventions than delayed breast reconstruction following PMRT and proved less time-consuming, even when the initial mastectomy is ignored. Therefore, we feel that the potential need of re-interventions should not be considered an impediment against such immediate reconstruction when a woman might be facing PMRT.

## Data Availability

The datasets generated during and/or analyzed during the current study are not publicly available due to legal restrictions but are available from the corresponding author upon reasonable request.

## Abbreviations

[N]SSM: Nipple- or skin-sparing mastectomy
IIBR: Immediate implant-based breast reconstruction
[N]SSM/IIBR: Nipple- or skin-sparing mastectomy with immediate implant-based breast reconstruction (combined surgery)
DBR: Delayed breast reconstruction
PMRT: Postmastectomy radiotherapy
IR group: Immediate reconstruction group
DR group: Delayed reconstruction group
